# Protection against H5N1 Influenza Virus Induced by Matrix-M Adjuvanted Seasonal Virosomal Vaccine in Mice Requires Both Antibodies and T Cells

**DOI:** 10.1371/journal.pone.0145243

**Published:** 2015-12-22

**Authors:** Freek Cox, Matthijs Baart, Jeroen Huizingh, Jeroen Tolboom, Liesbeth Dekking, Jaap Goudsmit, Eirikur Saeland, Katarina Radošević

**Affiliations:** Janssen Prevention Center, Center of Excellence of Janssen Research & Development, Pharmaceutical companies of Johnson and Johnson, Leiden, The Netherlands; Icahn School of Medicine at Mount Sinai, UNITED STATES

## Abstract

**Background:**

It remains important to develop the next generation of influenza vaccines that can provide protection against vaccine mismatched strains and to be prepared for potential pandemic outbreaks. To achieve this, the understanding of the immunological parameters that mediate such broad protection is crucial.

**Method:**

In the current study we assessed the contribution of humoral and cellular immune responses to heterosubtypic protection against H5N1 induced by a Matrix-M (MM) adjuvanted seasonal influenza vaccine by serum transfer and T-cell depletion studies.

**Results:**

We demonstrate that the heterosubtypic protection against H5N1 induced by MM adjuvanted vaccine is partially mediated by antibodies. The serum contained both H5N1 cross-reactive hemagglutinin (HA)- and neuraminidase (NA)-specific antibodies but with limited virus neutralizing and no hemagglutination inhibiting activity. The cross-reactive antibodies induced antibody-dependent cellular cytotoxicity (ADCC) in vitro, suggesting a role for the Fc part of the antibodies in protection against H5N1. Besides H5N1 specific antibody responses, cross-reactive HA- and NA-specific T-cell responses were induced by the adjuvanted vaccine. T-cell depletion experiments demonstrated that both CD4^+^ and CD8^+^ T cells contribute to protection.

**Conclusion:**

Our study demonstrates that cross-protection against H5N1 induced by MM adjuvanted seasonal virosomal influenza vaccine requires both the humoral and cellular arm of the immune system.

## Introduction

Human influenza infections are caused by influenza A, B and C viruses. Whereas influenza C infections are mild and generally clinically irrelevant, influenza A and B cause annual epidemics [1]. Currently, influenza A H1N1 and H3N2 subtypes and two influenza B strains, one from the Victoria-lineage and one from the Yamagata-lineage are circulating globally [2]. In addition, zoonotic influenza A strains, such as H5N1, can cross the species barrier and potentially cause pandemic outbreaks with high mortality rates [3,4].

Vaccination is considered the best way to prevent influenza related disease burden. The current seasonal influenza vaccines (containing antigens derived from an H1N1, an H3N2 and one or two influenza B strains) and pandemic vaccine candidates are mainly based on the hemagglutinin (HA), which is, together with the neuraminidase (NA), the major glycoprotein of the virus envelope. These types of vaccines aim to induce antibodies that target the receptor binding site located on the globular head of the HA molecule, thereby, blocking attachment of the viral HA to the sialic acid receptor on the host cell and consequently prevent infection. However, the HA head is very variable and therefore such antibodies only provide effective protection against closely matched strains [5,6]. In addition, many variants of different zoonotic viruses (including H5) circulate in animal hosts making it virtually impossible to predict which strain will break through the species barrier and cause the next pandemic in humans.

Therefore, there is an urgent need for influenza vaccines that induce broad reactive immunity and that can provide protection against mismatched seasonal and potential pandemic strains.

In order to realize such a broadly protective vaccine, it is important to understand what type of immune response is required for broad protection against influenza.

Broadly neutralizing monoclonal antibodies have been discovered that target the relatively conserved stem region of the HA molecule [7–10] and display potent prophylactic and therapeutic protective abilities in mice [7,8,10,11] and in ferrets [12]. Although broadly neutralizing monoclonal antibodies directed to the stem are able to directly neutralize influenza virus in vitro, there is evidence that they may require interactions with Fcγ receptors (FcγR) on immune cells to be effective in vivo [13]. Clearance of infected cells through FcγR-mediated effector function, such as antibody dependent cellular cytotoxicity (ADCC), adds an additional mechanism by which HA-specific antibodies can induce protection in vivo [14].

Next to HA-specific antibodies, antibodies against other viral proteins such as NA or the conserved matrix protein M2 may also confer heterologous protection [15–18]. NA-specific antibodies can prevent descendant viruses to egress and thereby inhibit viral spread and disease severity [19]. In addition, NA-specific antibodies can clear virus-infected cells via ADCC [14,20]. Like NA-specific antibodies, M2-specific antibodies do not prevent virus infection, but they have been shown to be protective via FcγR-mediated elimination of infected cells [21,22].

Although neutralizing antibodies are considered to be the main mechanism of protection against influenza [23], once infection is ongoing, T cells are likely to play a role in protection. In particular, CD8^+^ cytotoxic T cells (CTLs) are known to clear virus-infected cells via direct cytotoxic effects, while CD4^+^ T cells act either via similar mechanisms or indirectly by providing help to B cells and CTLs [24,25]. There is evidence that T cells directed to epitopes located in the relatively conserved internal proteins, such as the nucleoprotein (NP) and matrix protein M1, or to conserved epitopes in HA and NA, can cross-react with various subtypes of influenza A viruses and confer a level of heterosubtypic protection [26–35].

Recently, we have demonstrated that a seasonal trivalent virosomal vaccine (TVV) adjuvanted with the saponin-based adjuvant Matrix-M (MM) provides protection against highly pathogenic H5N1 virus in mice and reduces viral loads in the upper and lower respiratory tract of H5N1 challenged ferrets [36]. Furthermore, using serum transfer studies, Roos et al showed that antibodies induced by multiple immunizations with TVV can provide heterosubtypic protection [37]. Here we further evaluated the mechanism of protection in the mouse model and demonstrate that protection against H5N1 is independent of hemagglutination inhibiting antibodies, but requires H5N1-binding antibodies in combination with T cells.

## Results

### Serum from immunized animals confers partial protection against H5N1 in the absence of H5 HAI and virus neutralizing antibody titers

**Fig 1:**
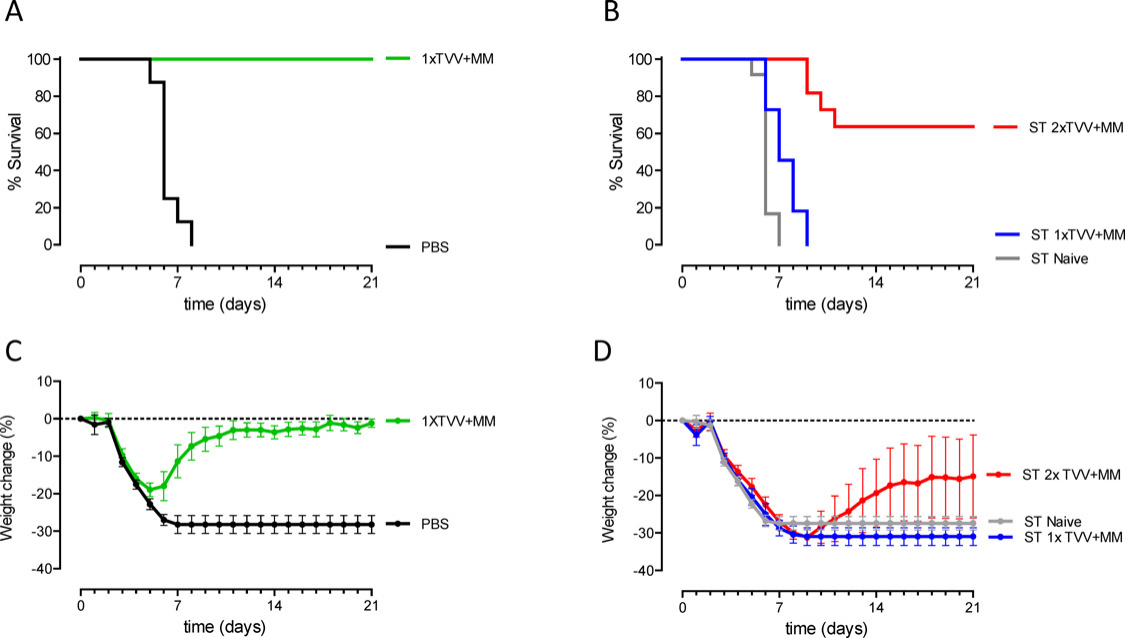
Serum antibodies confer partial protection against H5N1. (A) Mice (n = 8/group) were immunized once with TVV+MM or PBS as negative control 4 weeks before challenge. (B) Recipient mice (n = 11-12/group) received 400μl immune sera of 1-time or 2-times TVV+MM immunized donors or naïve serum intraperitoneally one day before challenge. Mice were challenged with 12.5xLD_50_ of wild type H5N1 A/Hong Kong/156/97 and monitored for 21 days for survival and weight-loss. Graphs A and B represent the Kaplan-Meier survival curves and graphs C and D represent mean bodyweight change with 95% confidence interval. ST = serum transfer.

To determine the contribution of antibodies to TVV+MM-induced protection against A/Hong Hong/156/97 (H5N1), serum pools were generated from groups of mice (donors) that were immunized once or twice with TVV+MM. A serum pool from naive donors was generated as a control. To confirm that the pools of immune serum contained antibodies that are able to provide protection we first tested these pools in a vaccine homologous H1N1 model. Mice were injected with the serum pools (recipients) or were actively immunized once with TVV+MM following challenge with vaccine homologous H1N1 virus. All recipients mice that received immune serum or mice that were actively immunized once with TVV+MM survived the lethal challenge while all mice that received naïve serum or were injected with PBS succumbed to the lethal challenge (S1A and S1B Fig). Subsequently, we investigated whether naïve recipients could be protected against lethal H5N1 challenge by the pooled immune sera in comparison to sera of naïve donors. Serum transfer efficiency was confirmed by assessing the vaccine homologous H1 specific antibody responses in serum of recipients (S1C Fig). We included mice that were immunized with TVV+MM or PBS as controls. The mice that were injected with PBS all succumbed, while mice that received a single immunization with TVV+MM survived the heterosubtypic H5N1 challenge and lost significant less bodyweight as compared to mice injected with PBS (p<0.001, Fig 1A and 1C). Recipients that received sera from donors immunized once with TVV+MM all succumbed to the H5N1 challenge; similar results were obtained for recipients that received naïve control sera (Fig 1B and 1D). However, 7 out of 11 (64%) recipients that received sera from donor mice that were immunized twice with TVV+MM survived the challenge (p<0.001 compared to recipients that received naïve sera) and showed less bodyweight-loss, although not significant as compared to recipients that received naïve sera (p = 0.103, Fig 1B and 1D).

### H5- and N1-binding antibody and ADCC responses are enhanced by Matrix-M adjuvation

**Fig 2:**
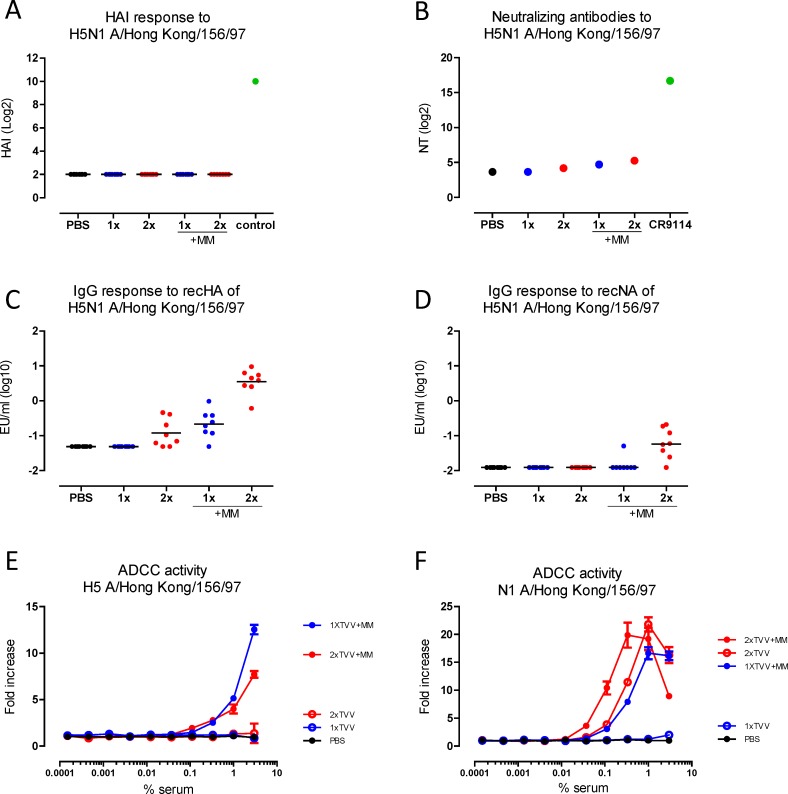
Cross-reactive HA and NA-specific ADCC responses are induced by TVV+MM. Mice (n = 8/group) were immunized once or twice with TVV or TVV+MM. Three weeks later, serum samples were obtained and tested for H5N1 A/Hong Kong/156/97 cross-reactive HAI responses (A), neutralizing antibody responses (B), HA-specific antibodies (C) and NA-specific antibodies (D) or ADCC responses against H5 expressing cells (E) or N1 expressing cells (F). Black bars indicate medians of log-2 transformed HAI and neutralizing titers (NT) or log-10 transformed ELISA titers (EU). Error bars in ADCC assays indicate the standard error of the duplicate means. Control in HAI assay = H5/HK specific sheep serum.

We investigated whether antibodies that prevent virus attachment as determined by a hemagglutination inhibition assay (HAI) may explain the observed protection against H5N1. However, HAI titers against H5N1 were not detected after immunization with TVV or TVV+MM (Fig 2A). Next, we measured neutralizing antibody titers against H5N1 using a sensitive pseudoparticle assay with pooled sera. Neutralizing antibody titers against H5N1 were low to non-detectable. However, serum pools from mice that received one or two immunizations with TVV+MM appeared to have higher titers than pools from mice receiving non-adjuvanted TVV (Fig 2B).

In the absence of HAI titers and high neutralizing antibody responses, we assessed whether sera of individual mice contained any H5N1 specific cross-reactive HA- or NA-binding antibodies. Whereas a single immunization with TVV alone did not result in detectable H5N1 HA-specific antibodies, a significant antibody titer was induced after two immunizations with TVV alone as compared to the vehicle control (p = 0.014). A single immunization with TVV+MM was sufficient to induce significant H5-specific antibodies levels compared to the vehicle control group (p = 0.003). H5-specific antibody titers were further increased after second immunization with TVV+MM (p = 0.001 compared to 1xTVV+MM, Fig 2C). Antibodies that target the conserved stem region of the HA, such as the human monoclonal antibody CR9114 [11], are known to display broad reactivity and may be a possible explanation for the observed H5-specific antibody response. Hence, we determined the levels of serum antibodies that could compete for binding with CR9114 as a measure for stem-directed antibodies. Stem-binding antibody titers, however, were not detected in serum of mice immunized with TVV or TVV+MM (S1D Fig).

Further, we assessed whether cross-reactive N1 (A/Hong Kong/156/97) antibody responses were induced after immunization with TVV or TVV+MM. While N1-specific antibody responses were neither observed after one or two immunizations with TVV alone nor after one immunization with TVV+MM, a significant induction of N1-specific antibody titers was induced after two immunizations with TVV+MM as compared to the vehicle control group (p = 0.003, Fig 2D).

Influenza-specific antibodies have previously been shown to mediate ADCC [14,20,21,38,39] and this may be one of the mechanisms by which antibodies mediate protection in our challenge model. Pooled sera were tested in a mouse-adapted ADCC surrogate assay that measures antigen-specific antibody-dependent FcγR activation. To measure ADCC responses to HA and NA separately, target cells were either transfected with HA or NA from H5N1 A/Hong Kong/156/97 before co-incubation with the reporter cell line (Jurkat cells expressing mouse FcγRIV, a major activating FcγR in mice [40]). HA-specific ADCC activity was observed in pooled serum from groups that received one or two immunizations with TVV+MM whereas ADCC activity was not detected in pooled serum from groups that received the non-adjuvanted vaccine or PBS (Fig 2E). NA-specific ADCC activity was detected after two immunizations with TVV alone or after one or two immunizations with TVV+MM, whereas no NA-specific activity was detected in pooled sera of animals that received a single injection with TVV alone or PBS (Fig 2F).

### Immunization with TVV+MM induces cross-reactive T-cell responses

**Fig 3:**
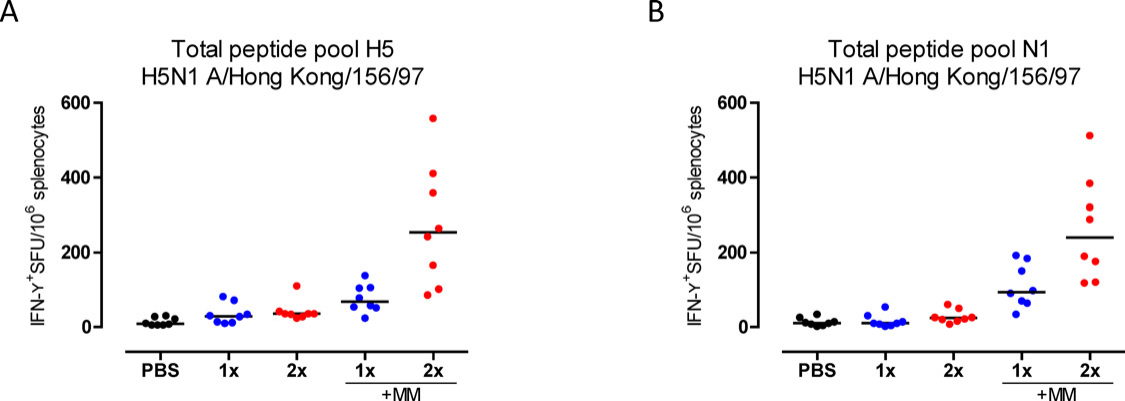
H5 and N1 cross-reactive T cells are induced by TVV+MM. Mice (n = 8/group) were immunized once or twice with TVV or TVV+MM. Three weeks later, spleens were harvested. The number of IFN-γ producing T cells was determined by ex vivo stimulation of splenocytes with peptide pools consisting of 15-mer peptides that cover the total (A) HA or (B) NA sequence of H5/HK with an 11-mer overlap. Black bars indicate medians of IFN-γ^+^ T cells per 10^6^ splenocytes. SFU = Spot forming units.

The antibody responses induced by TVV+MM only partially protected mice against H5N1 virus. To identify other possible mechanisms of protection, we determined the levels of HA and NA specific cross-reactive interferon-gamma (IFN-γ) producing T cells after immunization with TVV or TVV+MM. One immunization with TVV alone did not result in significant induction of H5-specific IFN-γ producing T cells compared to the vehicle control group (p = 0.077). However, significant levels of IFN-γ producing T cells were detected after two immunizations with TVV or one immunization with TVV+MM (p = 0.008 and p<0.001, respectively, compared to vehicle control). The T-cell responses were enhanced by MM adjuvation compared to 1xTVV or 2xTVV (both p<0.001) (Fig 3A). Similarly, significant levels of cross-reactive N1-specific IFN-γ producing T cells were induced after immunization with 1xTVV+MM or 2xTVV+MM (p<0.001 for both, compared to vehicle control) (Fig 3B).

Seasonal influenza vaccines are known to contain traces of internal proteins like M1, M2, and NP in addition to HA and NA, and these may elicit cross-reactive T-cell responses [41]. In our study, a low but detectable NP-specific IFN-γ producing T-cell response was detected after 1xTVV+MM or 2xTVV+MM (p<0.001 for both, compared to vehicle control) (S2A Fig). However, a T-cell response directed to the immune dominant H-2d restricted NP_147-155_ specific CD8 epitope was not detected (S2B Fig). No statistically significant levels of M1- or M2- specific IFN-γ producing T cells were detected, indicating that it is unlikely that these T cells play a role in heterosubtypic protection in our model (S2C and S2D Fig).

### CD4^+^ and CD8^+^ T cells both contribute to H5N1 protection

To investigate whether the cross-reactive T-cell responses play a role in TVV+MM induced protection, CD4^+^, CD8^+^ or both CD4^+^ and CD8^+^ T cells were depleted in vaccinated mice by specific monoclonal antibody injections four days and one day before challenge. To confirm comparable antibody responses after vaccination in all groups, we first assessed the antibody titers against recH1 as a measure for response to vaccine. As shown in S3A and S3B Fig, all groups of mice showed comparable HA-specific antibody responses.

**Fig 4:**
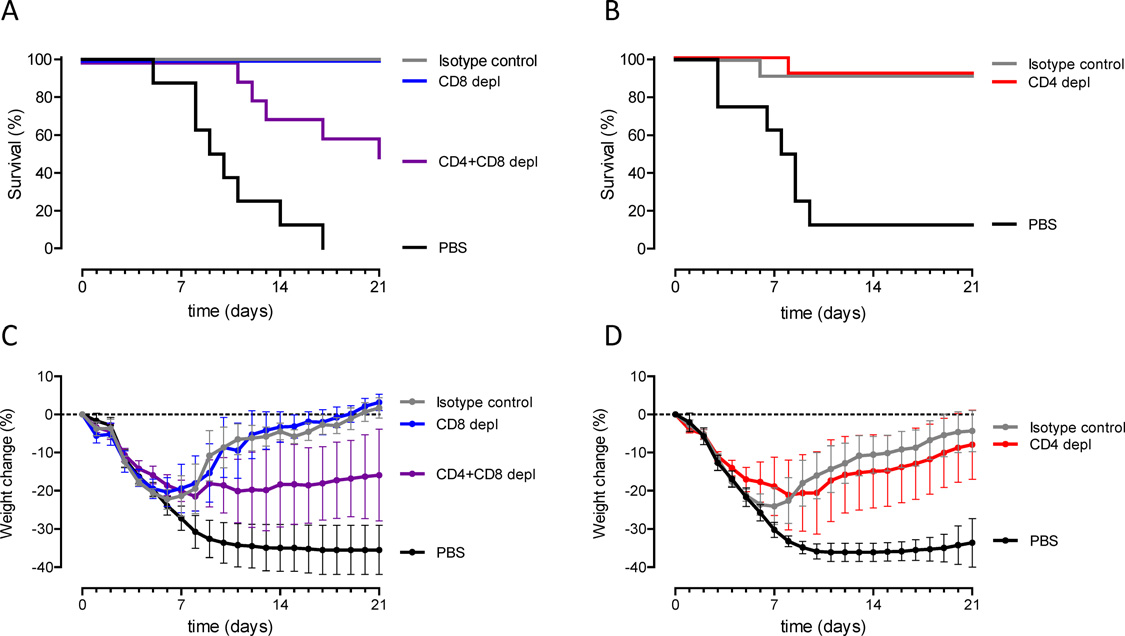
CD4^+^ and CD8^+^ T cells both contribute to H5N1 protection. Mice (n = 8-10/group) were immunized once with TVV+MM or PBS as negative control 4 weeks before challenge and (A) CD8^+^ or the combination of CD4^+^ and CD8^+^ T cells were depleted or (B) CD4^+^ T cells only were depleted with antibodies injected 4 days and 1 day before challenge. Mice were challenged with 12.5xLD_50_ of wild type H5N1 A/Hong Kong/156/97 and monitored for 21 days for (A and B) survival and (C and D) bodyweight-loss. Graphs A and B represent the Kaplan-Meier survival curves and graphs C and D represent mean bodyweight change with 95% confidence interval.

The depletion of CD4^+^ or CD8^+^ separately did not result in significantly reduced survival proportion or decreased bodyweight-loss compared to mice that were treated with an isotype control antibody (Fig 4A–4C). However, only 50% of the mice survived the lethal H5N1 challenge when both CD4^+^ and CD8^+^ T cells were depleted compared to 100% survival of isotype injected control animals (p = 0.033, Fig 4A). In addition, CD4^+^ and CD8^+^ T cells depleted mice showed decreased bodyweight-loss compared to isotype injected control animals (p = 0.08). These results indicate that both CD4^+^ and CD8^+^ T cells contribute to TVV+MM induced protection against H5N1.

## Discussion

It remains important to develop the next generation of influenza vaccines that can provide protection against vaccine mismatched strains and to be prepared for potential pandemic outbreaks. One of the approaches to induce broadly protective immune responses against influenza is adjuvation of current seasonal vaccines. Matrix-M has pronounced immunopotentiating properties and has been evaluated in combination with a pandemic virosomal influenza vaccine in mice [42,43] and humans [44,45]. In both species, the immunopotentiating activities were characterized by enhancement of humoral and cellular immune responses [42–45]. We have recently shown that adjuvation of TVV improved protection in mice against vaccine heterologous H1N1, H3N2 and influenza B strains (Cox et al, 2015, in press, Virology Journal). In addition, when adjuvanted with MM, TVV even induced protection against avian H5N1 and H7N7 strains in mice and provided partial protection against H5N1 in ferrets [36]. Understanding the immunological mechanisms that provide heterologous and heterosubtypic protection is important since this knowledge will eventually lead to design of better seasonal and pandemic influenza vaccines. In the current study we investigate the mechanism of protection against H5N1 in mice induced by immunization with MM adjuvanted TVV and show that protection against H5N1 requires both antibody and T-cell responses.

Mice were fully protected against lethal H5N1 challenge even after a single immunization with TVV+MM. However, passive transfer of serum from mice that received two immunizations with TVV+MM yet only partly protected the recipients, indicating that antibodies contribute to protection against H5N1 but that they are not the sole mediator. Currently, antibodies that prevent the HA from interacting with the host cell are the only accepted in vitro correlate of protection and they can be measured by the HAI assay. In our study HAI responses against H5N1 were not detected, but the adjuvation of TVV with MM induced H5N1-specific cross-reactive HA- and NA-specific antibody responses, and low but detectable H5N1 neutralizing antibody responses. Although the neutralizing antibody responses were low in an in vitro assay, they are likely to contribute to a certain level of protection against H5N1. It has been reported previously that ADCC plays a role in heterologous protection against influenza [21,22,32,34,46,47]. We demonstrated that the ADCC responses against HA and NA were enhanced by MM adjuvation. Noteworthy, the NA-specific ADCC responses were more potent than the HA-specific ADCC responses despite the fact that the total HA-specific antibody responses, as measured in ELISA, were higher in comparison to the NA-specific antibody responses. The sensitivity between the assays may most likely contribute to observed differences, however our data suggests that the NA-specific FcγR-mediated effector function may be an important immunological mechanism involved in the protection against H5N1. In agreement, several studies have been shown that NA-specific antibodies are associated with heterologous protection in both mice and ferrets by means of ADCC, but other mechanisms such as inhibition enzymatic activity of NA may play a role as well [15–17,48].

As mentioned above, antibodies are likely not the sole mediators of protection in our model. Here we demonstrate that T cells contribute to the protection against H5N1 induced by TVV+MM as well. Depletion of CD4^+^ and CD8^+^ T cells before challenge significantly reduced the survival proportion (50% survival) of immunized mice, thereby demonstrating that both CD4^+^ and CD8^+^ T cells contribute to protection. However, depletion of either CD8^+^ or CD4^+^ before the challenge had no effect on survival proportion or bodyweight-loss and clinical scores (data not shown) of TVV+MM immunized mice compared to control animals. These data demonstrate that the presence of only one of the T-cell subsets is sufficient for full protection in combination with serum antibodies that are present.

CD8^+^ T cells directed against the conserved internal proteins such as nucleoprotein (NP) and matrix protein (M1), in particular, have shown to have beneficial effects on the course of infection in mice [49–51]. In humans, CD4^+^ T cells correlate to protection in the absence of virus specific antibodies [52], but virus specific CD8^+^ T cells were shown to correlate with cross-protection against symptomatic pandemic H1N1 [53]. In our study, the major cross-reactive T-cell responses were directed against the HA and NA protein, but also some NP-specific T-cell responses were detected. T cells directed against the M1 and M2 proteins were not detected after immunization with TVV+MM and the relative contribution of these T cells to protection remains to be elucidated.

In agreement with our results, an experimental monovalent H1N1-based vaccine formulated with saponin-based immune stimulating complexes (ISCOMs) has been shown to induce heterosubtypic protection in mice [31,32]. The protection was accompanied by severe disease symptoms as evidenced by bodyweight-loss. The protection was independent of HAI reactive antibodies but it required cross-reactive binding antibodies in combination with HA-specific CD8^+^ responses. The cross-reactive antibodies were able to reduce virus infection in the presence of macrophages in an in vitro cell-based assay [31,32].

In conclusion, our study demonstrates that protection against H5N1 induced by Matrix-M adjuvanted trivalent virosomal vaccine requires both antibodies and T cells and support the notion that different immunological mechanism may play a role in protection against influenza depending on the vaccine type.

## Materials and Methods

### Statement of Ethics

All mouse experiments were performed in accordance with Dutch legislation on animal experiments and approved by an independent institutional review board (DEC- “Dier Experimenten Commissie”).

### Immunization

Six-to-eight-week-old female BALB/c (H-2d) mice (specific pathogen-free) were purchased from Charles River laboratories (Sulzfeld, Germany). H1N1 A/California/07/09, H3N2 A/Victoria/210/09 and B/Brisbane/60/08 monovalent virosomes were prepared by Crucell (Berne, Switzerland) using conventional procedures [54]. The monovalent virosomes were mixed to obtain a trivalent virosomal vaccine (TVV, 3μg HA per strain). Matrix-M (MM, 10μg/dose, Novavax AB, Uppsala, Sweden) was mixed with TVV before immunization (TVV+MM). Mice were immunized once or twice with TVV or TVV+MM via the intramuscular (i.m.) route 3 weeks apart with 100μl vaccine (50μl per hind leg). Control groups received 100μl PBS. Three weeks after the final immunization serum and spleens were collected to assess humoral and cellular responses, respectively.

### Influenza challenge

Four weeks after the final immunization mice were anesthetized by intraperitoneal (i.p.) administration of 100mg/kg ketamine (Nimatek 100mg/ml, Eurovet, Cuijk, the Netherlands) in combination with 20mg/kg xylazine (Sedamun 20mg/ml, Eurovet). Mice were challenged with (i) 25xLD_50_ of H1N1 A/Netherlands/602/09 (performed at Janssen Research and Development, Leiden, the Netherlands) or (ii) 12.5xLD_50_ wild type H5N1 A/Hong Kong/156/97 (performed at CVI Lelystad, the Netherlands) via the intranasal route (a total of 50μl, 25μl per nostril). After challenge, mice were monitored for bodyweight-loss, clinical score (data not shown) and survival for 21 days or until humane endpoint.

Our experience with Influenza challenge models suggests that using alternative humane endpoints, such as bodyweight-loss would lead to underestimation of protection. The amount of bodyweight-loss before animals reach clinical score 4 is variable. Therefore we used clinical score 4 as a humane endpoint to accurately assess protection by vaccination. We explicitly discussed this issue with our Ethical Review Board, and they agreed to allow clinical score 4 as a humane endpoint. The clinical scores for challenges were defined as: 0 = no clinical signs, 1 = rough coat, 2 = rough coat, less reactive, passive during handling, 3 = rough coat, rolled up, labored breathing, passive during handling, 4 = rough coat, rolled up, labored breathing, unresponsive (= humane endpoint) or found dead (= score 4). Mice that died before reaching humane endpoint were scored as clinical score 4. Mice that reached humane endpoint were sacrificed by cervical dislocation under isoflurane anesthesia as well as mice that were still alive at the end of the study.

### Serum transfer

To generate immune serum groups of mice (n = 50) were immunized once or twice with TVV+MM. Four weeks after final immunization blood was collected via heart puncture under isofluorane (IsoFlo, Abbott Park, IL, USA) anesthesia followed by cervical dislocation. Serum was collected after centrifugation for 4 minutes at 1699×*g* followed by 1 minute at 20817×*g*. Mice (n = 12) were injected intraperitoneally (i.p.) with 400μl immune serum pool or naïve serum pool one day before challenge. The levels of vaccine homologous H1-specific antibodies in recipients were determined at the day of challenge as a measure of serum transfer efficiency (S1C Fig).

### Hemagglutination Inhibition (HAI) assay

HAI assays were performed as described before [42]. Briefly, sera were pre-absorbed with 0.5% turkey red blood cells (bioTRADING Benelux B.V., Mijdrecht, the Netherlands) in PBS for 2 hours. After removal by centrifugation, sera were treated for 16 hour with a receptor-destroying enzyme (Sigma-Aldrich; St. Louis, MO, USA; diluted 1:25 in PBS). The receptor-destroying enzyme was heat inactivated for 30 minutes at 56°C. Two-fold serial dilutions of the sera (initial dilution 1:8) or a positive control (Influenza Antiserum H5/HK, NIBSC, London, England) were incubated for 1 hour at room temperature with reassortant H5N1 A/Hong Kong/156/97 standardized to 8 hemagglutination units. Turkey red blood cells were added and incubated for 60 minutes before hemagglutination inhibition was determined. Each serum sample was tested in duplicate. Titers were expressed as the reciprocal of the highest dilution where complete agglutination inhibition was observed.

### HA-and NA-based ELISA

To assess HA- and NA-specific binding antibody levels in serum samples, Recombinant HA (recHA) of H1N1 A/California/07/09 (Protein Sciences Inc., CT, USA) or recHA or recNA of H5N1 A/Hong Kong/156/97 (Both produced on HEK293F cells) were coated at an concentration of 0.5μg/ml onto Maxisorp 96-well plates (Nunc, Thermo Scientific) O/N at 4°C. Plates were washed with PBS (Life Technologies, Paisley, UK) containing 0.05% Tween-20 (Merck Millipore, Darmstadt, Germany) (PBS-T) and subsequently blocked with PBS containing 2% dried skimmed milk (BD, Breda, the Netherlands) for the HA-based ELISA or with PBS containing 2% bovine serum albumin (BSA; Sigma) (PBS/BSA) for the NA-based ELISA for 1 hour at RT. Following a wash with PBS-T, serum of individual mice were added to the plate in duplicate, serially diluted (2-fold, 0.002–2%) and incubated for 1 hour at RT. Following a wash with PBS-T a 1:2000 dilution of goat-anti-mouse HRP conjugated (KPL, Maryland, USA) was added to the plate and incubated for 1 hour at RT. After washing with PBS-T, OPD substrate (Thermo Scientific) was added to the plate. The colorimetric reaction was stopped after 10 minutes by adding 1M H_2_SO_4_. The optical density (OD) was measured at 492 nm and standard curves were created using a four parameter logistic curve. The OD of each sample dilution was then quantified against the standard (HA standard: human CR9114 with a mouse IgG2a Fc-part, NA standard: mouse Monoclonal Antibody, clone 6G6 (Immune Technology Corp., New York, USA)) and the final concentration per sample (in ELISA Units, EU/ml) calculated by a weighted average, using the squared slope of the standard curve at the location of each quantification as weight. Negative samples were set at the limit of detection (LOD), defined as the lowest sample dilution multiplied by the lowest standard concentration with an OD response above the lower asymptote of the standard curve and background.

### CR9114 competition ELISA

To measure antibody responses competing with the human IgG1 CR9114 monoclonal antibody binding to a conserved epitope in the stem region of influenza HA [11], a modified ELISA protocol was used. Maxisorp 96-well plates (NUNC) were coated with purified polyclonal rabbit anti His-Tag IgG (Genscript USA Inc., NJ, US) O/N at 4°C followed by washing. After blocking with PBS/BSA and washing, plates were incubated with a titrated amount of His-Tagged HA of A/Brisbane/59/07 (produced in HEK293F cells) for 2 hours at RT. Plates were washed and individual serum samples were added to the plate in duplicate, serially diluted in PBS/BSA and incubated for 1 hour at RT, followed by addition of a titrated amount of biotinylated human CR9114 and incubation for another hour at RT. After washing, streptavidin-HRP was added and incubated for 1 hour at RT, followed by washing and OPD development. The CR9114 competition of each sample was quantified as the slope of the linear regression of OD value on the log10 dilution for the duplicate series. Positive and negative controls consisted of competition with monoclonal antibodies CR9114 (at 2.5μg/ml) [11].

### Pseudoparticle neutralization assay

Pseudoparticles expressing HA were generated as previously described [55]. Neutralizing antibodies were determined using a single transduction round of HEK293 cells with H5 A/Hong Kong/156/97 pseudoparticles encoding luciferase reporter gene, as described previously [56], with a few modifications. Briefly, heat-inactivated (30 minutes at 56°C) serum samples were 3-fold serially diluted in growth medium (MEM Eagle with EBSS (Lonza, Basel, Switzerland)) supplemented with 2 mM L-Glutamine (Lonza), 1% Non-Essential Amino Acid Solution (Lonza), 100 U/ml Pen/Strep (Lonza) and 10% FBS (Euroclone, Pero, Italy)) in triplicate in 96-well flat bottom culture plates and a titrated number of H5 A/Hong Kong/156/1997 (yielding ~100 relative luminescence units (RLU) post-infection) was added. After 1 hour of incubation at 37°C, 5% CO2 10^4^ HEK293 cells were added per well. After 48 hours of incubation at 37°C, 5% CO2 luciferase substrate (Britelie Plus, Perkin Elmer, Waltham, US) was added and luminescence was measured using a luminometer (Mithras LB 940, Berthold Technologies, Germany) according to manufacturers’ instructions. A mouse version of monoclonal antibody CR9114 (mouse IgG2a, produced in house) was used as a positive control. To calculate the EC50 value the Spearman-Kärber method was used.

### Antibody Dependent Cellular Cytotoxicity (ADCC) assay

Human lung carcinoma–derived A549 epithelial cells (ATCC CCL-185) were maintained in Dulbecco's modified eagle medium (DMEM) supplemented with 10% heat inactivated fetal calf serum (Gibco) at 37°C, 10% CO2. Two days before the experiment, A549 cells were transfected with plasmid DNA encoding for H5 or N1 of A/Hong Kong/156/97 using Lipofectamine 2000 (Invitrogen) in Opti-MEM (Invitrogen). One day before the assay, transfected cells were harvested and seeded in white 96-well plates (Costar). After 24 hours, samples were diluted in assay buffer (4% ultra-low IgG FBS (Gibco) in RPMI 1640 (Gibco)) and heat inactivated for 30 minutes at 56°C, followed by serial dilution in assay buffer. The cells were replenished with fresh assay buffer and antibody dilutions and ADCC Bioassay effector cells (a stable Jurkat cell expressing mouse Fc gamma receptor IV (FcγRIV), human CD3γ, and an NFAT- response element regulating a luciferase reporter gene [40]), were added and incubated for 6 hours at 37°C at a target-effector ratio of 1:4.5. Cells were equilibrated to room temperature for 15 minutes before Bio-Glo Luciferase System substrate (Promega, Madison, US) was added. Luminescence was read out after 10 minutes on a Synergy Neo (Biotek, Winooski, US). A mouse version of monoclonal antibody CR9114 (mouse IgG2a, produced in house) was used as a positive control, (data not shown). Data is shown as fold increase over background.

### IFN-γ producing T cells (ELISPOT)

To assess influenza specific T-cell responses splenocytes were ex-vivo stimulated with total peptide pools, consisting of 15-mers with 11 amino acids overlap of: HA and NA of H5N1 A/Hong Kong/156/97; M1 and M2 of H1N1 A/California/07/09; NP H3N2 A/Kitakyushu/159/1993 or an immune dominant H-2d restricted NP specific CD8 epitope, NP_147-155_ (JPT, Berlin, Germany).

Splenocytes were prepared in R10 medium (RPMI 1640, Invitrogen, The Netherlands) containing 10% heat inactivated FBS (HyClone, Perbio Science Nederland BV), 1% Pen/Strep (Invitrogen), 1% MEM non-essential amino acids (Invitrogen) and 13μM 2-mercaptoethanol (Fluka Chemie, Switzerland). Anti-IFN-γ precoated 96-wells PVDF plates (DIACLONE, Ann Arbor, US) were washed once with DPBS pH7.4 (Invitrogen) before 5x10^5^ splenocytes per well were added in duplicate with 2ug/ml per peptide, or medium as negative control. After 18 h incubation at 37°C, 10% CO2, cells were removed by washing with D-PBS/Tween. Plates were then incubated for 1.5 hours at room temperature with biotinylated Rat-anti-mouse-IFN-γ (DIACLONE). Next, plates were washed and incubated for 1.5 hours at room temperature with alkaline phosphatase conjungated streptavidin (DIACLONE). After final washing, specific spot formation was developed by incubating the plates for 10 minutes with BCIP/NBT substrate (DIACLONE). The colorimetric reaction was stopped by washing the plates with tab water. After air-drying the number of spots was quantified with an AELVIS ELISPOT reader (AELVIS GmbH, Sanquin, the Netherlands).

### T-cell depletion

CD4^+^ or CD8^+^ T cells were depleted by i.p. injections of 100μg functional grade rat-anti-mouse-CD4 clone GK1.5 (eBioscience, San Diego, US), rat-anti-mouse CD8a clone 2.43 (Leinco technologies, St. Louis, US), a combination of both to deplete CD4^+^ and CD8^+^ T cells or matched rat isotype control (eBioscience) 4 days and 1 day before challenge. At the day of challenge, ~100 μl blood was collected in heparin-coated tubes (Sarstadt, Nümbrecht, Germany) to assess depletion efficacy by flow cytometry (data not shown). CD4^+^ T cells were defined as CD3^+^CD4^+^CD8^-^ cells and CD8^+^ T cells were defined as CD3^+^CD4^-^CD8^+^ cells within the lymphocyte population. No mice were excluded from analysis based on the predefined exclusion criteria of less than 70% depletion efficacy. Plasma of the corresponding samples was used to asses vaccination efficacy by the recH1 ELISA as described.

### Statistical analysis

Statistical differences between immunizations with TVV with or without Matrix-M relative to negative control group receiving PBS were evaluated for HAI titers, HA and NA-specific binding antibodies, CR9114-competing antibodies and the different stimulations in the ELIspot assay. Data were log-transformed except for the competition ELISA and comparisons between groups were made using either t-test in ANOVA or the Wilcoxon rank-sum test with adjustment for multiple comparisons (2-fold Bonferroni and for comparisons with PBS a stepwise approach testing first 2x and then 1x vaccination). Additionally, the effect of two immunizations compared to one immunization and the effect of Matrix-M compared to TVV alone were determined (S1 Table and S2 Table) using either t-test in ANOVA or the Wilcoxon rank-sum test with adjustment for multiple comparisons (4-fold Bonferroni).

In the serum transfer studies the groups that received immune sera were compared to the vehicle control group receiving naïve serum for survival proportion, change in bodyweight and clinical scores. To assess the effect of depleting CD8^+^ T cells or both CD8^+^ and CD4^+^ T cells, depleted group were compared to the matching isotype control in a stepwise approach starting with the CD8^+^ and CD4^+^ depleted group. The effect of depletion of CD4^+^ T cells was performed in a separate experiment. The CD4^+^ T cells depleted group was compared to matching isotype treated group for survival proportion, change in bodyweight and clinical scores. For survival proportion after challenge, a Fisher’s exact test was performed. For bodyweight-loss analysis, repeated measurements in the challenge phase were summarized as a single outcome per animal using an Area Under The Curve (AUC) approach where missing values for animals that died before day 21 were imputed with a last-observation-carried-forward method. Bodyweight data are expressed as the change relative to the day 0 measurement. The AUC was defined as the summation of the area above and below the baseline. An ANOVA on AUC’s was performed with group as explanatory factor.

Statistical analyses were performed using SAS version 9.4 (SAS Institute Inc. Cary, NC, US) and SPSS version 20 (SPSS Inc., IL, US). Statistical tests were conducted two-sided at an overall significance level of α = 0.05.

## Supporting Information

S1 Fig Serum characterization and serum transfer efficiency. Recipients (n = 11-12/group) received 400μl immune sera of 1-time or 2-times TVV+MM immunized donors or naïve serum one day before challenge (A) or mice (n = 8/group) were immunized once with TVV+MM or PBS as negative control 4 weeks before challenge (B). Mice were challenged with 25xLD_50_ of vaccine homologous A/Netherlands/602/09 and monitored for 21 days for survival. Graphs (A and B) represent the Kaplan-Meier survival curves. C) Serum samples of mice that were actively immunized or that received immune or naïve control serum following H5/HK challenge were isolated at the day of challenge to determine H1-specific (recHA of A/California/07/09) antibody responses that were compared to the original pools before transfer. D) The presence of CR9114-competing antibodies serum of mice that received 1 or 2-times TVV with or without MM or PBS was assessed and depicted as slope OD values as described in the material and method section. Black bars indicate medians of log-10 transformed ELISA titers (EU) or median slope OD values in case of the CR9114 competition ELISA.
(TIFF)

S2 Fig Low/absent T-cell responses to internal proteins or M2 after immunization. Mice (n = 8/group) were immunized once or twice with TVV or TVV+MM. Three weeks later, spleens were harvested. The number of IFN-γ producing T cells was determined by ex vivo stimulation of splenocytes with peptide pools consisting of 15mers peptides that cover the total sequence with 11mer overlap (= total peptide pool) of NP of H3N2 (Swiss-Prot ID: O91743) (A), M1 (Swiss-Prot ID: C3W5Z8) (C) or M2 (Swiss-Prot ID: C3W5Z7) (D) or stimulated with a H2-d dominant NP-specific CD8^+^ T-cell epitope (GenBank: AAM75159.1) (B). Black bars indicate medians of IFN-γ^+^ T cells per 10^6^ splenocytes.
(TIFF)

S3 Fig Pre-challenge antibody responses were comparable between different groups. Mice (n = 8-12/group) were immunized once with TVV+MM or PBS as negative control 4 weeks before challenge. (A) CD8^+^ or the combination of CD4^+^ and CD8^+^ T cells or (B) CD4^+^ T cells were depleted with depleting antibodies or matching isotype control injections 4 days and 1 day before challenge. At the day of challenge plasma samples were isolated to determine H1-specific (recHA of A/California/07/09) antibody responses. Black bars indicate medians of log-10 transformed ELISA titers (EU).
(TIFF)

S1 Table Summarizes p-values of various group comparisons of antibody responses. Statistical analysis was performed as described in the material and methods section. TVV = Trivalent Virosomal Vaccine. MM = Matrix-M. nd = no statistical analyses performed.
(TIF)

S2 Table Summarizes p-values of various group comparisons of T-cell responses. Statistical analysis was performed as described in the material and methods section. TVV = Trivalent Virosomal Vaccine. MM = Matrix-M. TP = Total peptide pool. nd = no statistical analyses performed.
(TIF)
