# Supplementary figures and images for: Protection against H5N1 Influenza Virus Induced by Matrix-M Adjuvanted Seasonal Virosomal Vaccine in Mice Requires Both Antibodies and T Cells
Source: PLoS One. 2015 Dec 22;10(12):e0145243. doi: 10.1371/journal.pone.0145243 (PMC4687931; doi:10.1371/journal.pone.0145243)

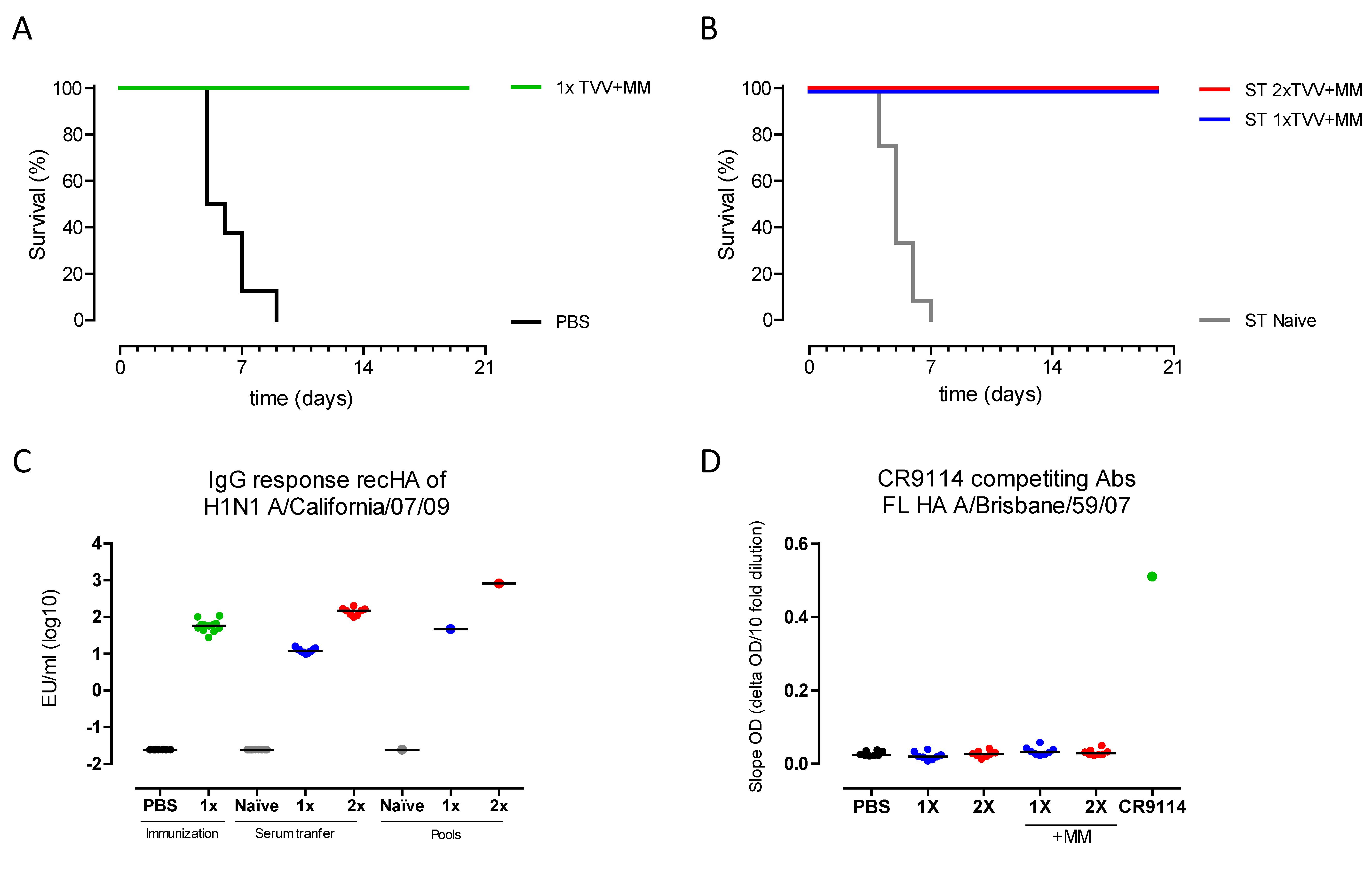

Supplement: S1 Fig — Recipients (n = 11-12/group) received 400μl immune sera of 1-time or 2-times TVV+MM immunized donors or naïve serum one day before challenge (A) or mice (n = 8/group) were immunized once with TVV+MM or PBS as negative control 4 weeks before challenge (B). Mice were challenged with 25xLD50 of vaccine homologous A/Netherlands/602/09 and monitored for 21 days for survival. Graphs (A and B) represent the Kaplan-Meier survival curves. C) Serum samples of mice that were actively immunized or that received immune or naïve control serum following H5/HK challenge were isolated at the day of challenge to determine H1-specific (recHA of A/California/07/09) antibody responses that were compared to the original pools before transfer. D) The presence of CR9114-competing antibodies serum of mice that received 1 or 2-times TVV with or without MM or PBS was assessed and depicted as slope OD values as described in the material and method section. Black bars indicate medians of log-10 transformed ELISA titers (EU) or median slope OD values in case of the CR9114 competition ELISA. (TIFF) [file pone.0145243.s001.tiff]

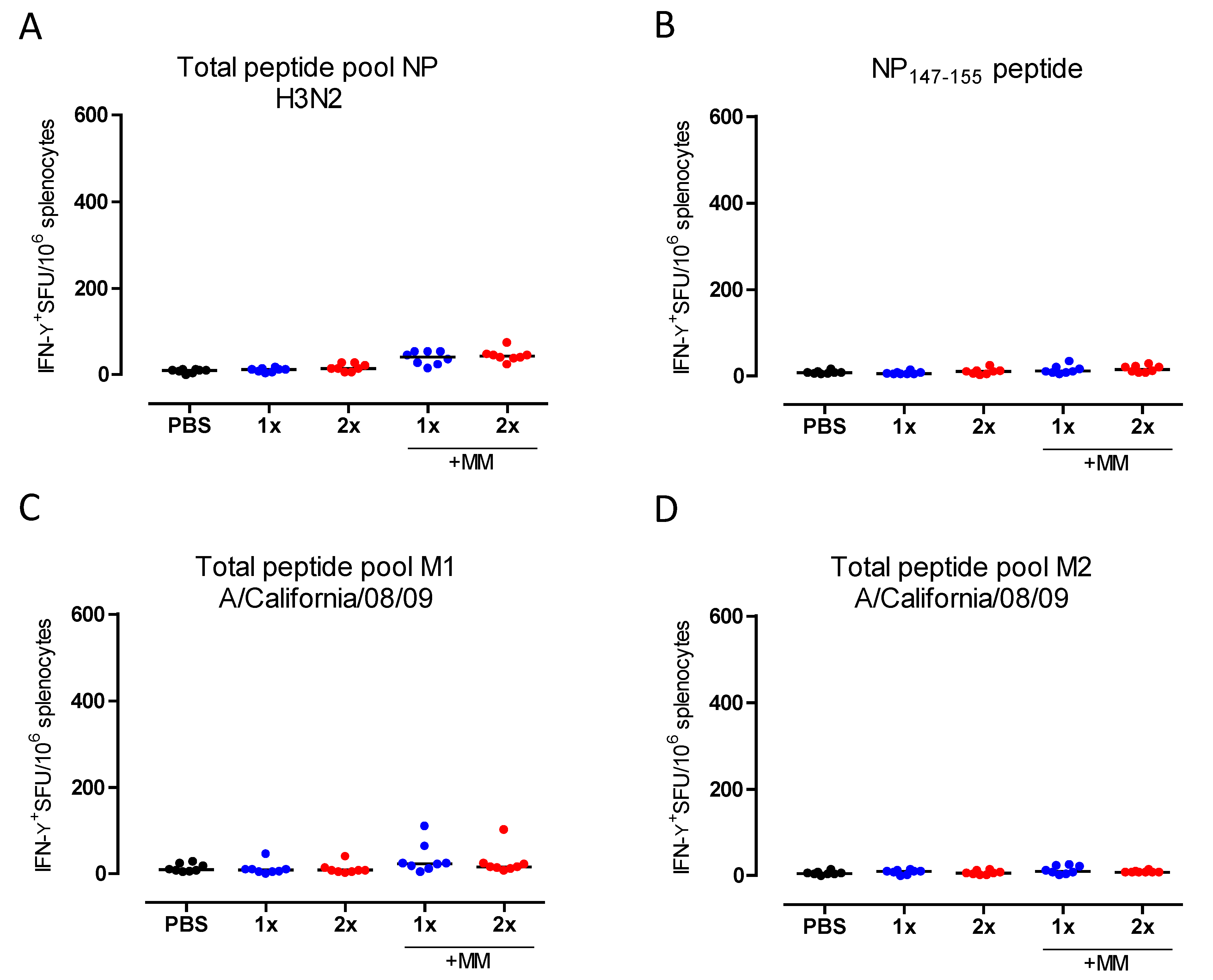

Supplement: S2 Fig — Mice (n = 8/group) were immunized once or twice with TVV or TVV+MM. Three weeks later, spleens were harvested. The number of IFN-γ producing T cells was determined by ex vivo stimulation of splenocytes with peptide pools consisting of 15mers peptides that cover the total sequence with 11mer overlap (= total peptide pool) of NP of H3N2 (Swiss-Prot ID: O91743) (A), M1 (Swiss-Prot ID: C3W5Z8) (C) or M2 (Swiss-Prot ID: C3W5Z7) (D) or stimulated with a H2-d dominant NP-specific CD8+ T-cell epitope (GenBank: AAM75159.1) (B). Black bars indicate medians of IFN-γ+ T cells per 106 splenocytes. (TIFF) [file pone.0145243.s002.tiff]

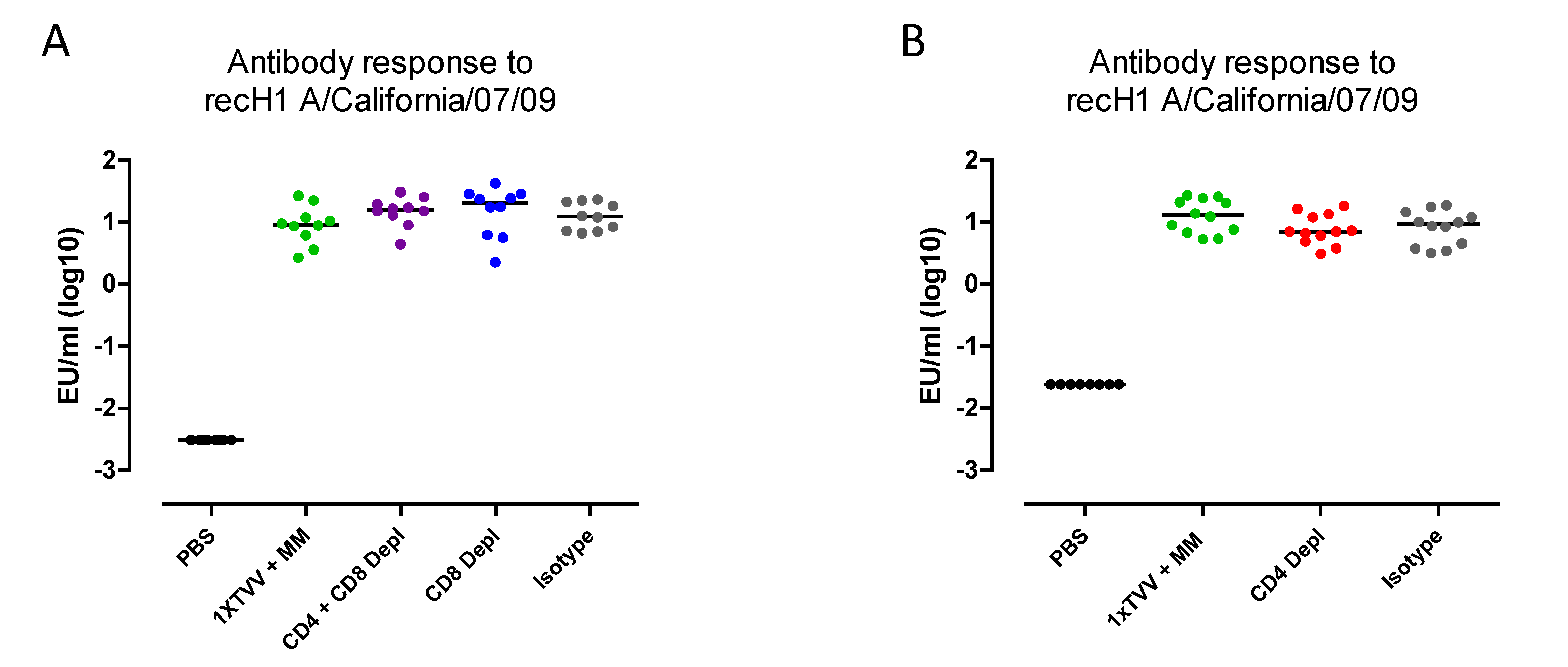

Supplement: S3 Fig — Mice (n = 8-12/group) were immunized once with TVV+MM or PBS as negative control 4 weeks before challenge. (A) CD8+ or the combination of CD4+ and CD8+ T cells or (B) CD4+ T cells were depleted with depleting antibodies or matching isotype control injections 4 days and 1 day before challenge. At the day of challenge plasma samples were isolated to determine H1-specific (recHA of A/California/07/09) antibody responses. Black bars indicate medians of log-10 transformed ELISA titers (EU). (TIFF) [file pone.0145243.s003.tiff]

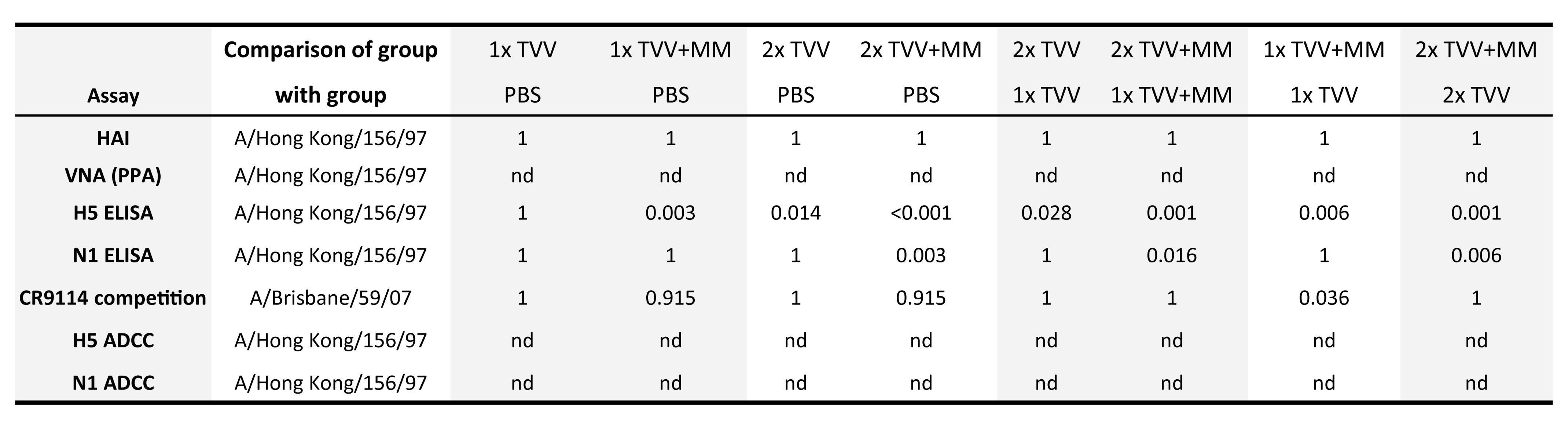

Supplement: S1 Table — Statistical analysis was performed as described in the material and methods section. TVV = Trivalent Virosomal Vaccine. MM = Matrix-M. nd = no statistical analyses performed. (TIF) [file pone.0145243.s004.tif]

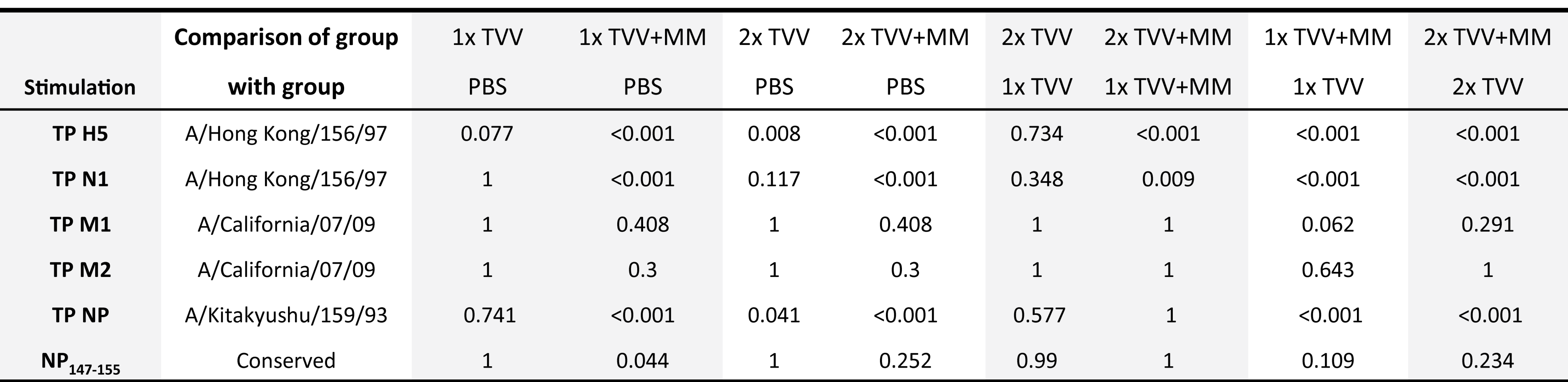

Supplement: S2 Table — Statistical analysis was performed as described in the material and methods section. TVV = Trivalent Virosomal Vaccine. MM = Matrix-M. TP = Total peptide pool. nd = no statistical analyses performed. (TIF) [file pone.0145243.s005.tif]
